# *TET2* rs1548483 SNP Associating with Susceptibility to Molecularly Annotated Polycythemia Vera and Primary Myelofibrosis

**DOI:** 10.3390/jpm10040259

**Published:** 2020-12-01

**Authors:** Diana L. Lighezan, Anca S. Bojan, Mihaela Iancu, Raluca M. Pop, Ștefana Gligor-Popa, Florin Tripon, Adriana S. Cosma, Ciprian Tomuleasa, Delia Dima, Mihnea Zdrenghea, Bogdan Fetica, Ioana Ioniță, Ildikó O. Gaál, Simona Vișan, Andreea-Manuela Mirea, Radu A. Popp, Mira Florea, Cătălin Araniciu, Lucian Petrescu, Ioan V. Pop, Claudia Bănescu, Adrian P. Trifa

**Affiliations:** 1 Department of Hematology, “Victor Babeș” University of Medicine and Pharmacy, 2, Eftimie Murgu Square, 300041 Timișoara, Romania; lighezan.diana@umft.ro (D.L.L.); mdioanaionita@yahoo.com (I.I.); 2 Department of Hematology, The Oncology Institute “Ion Chiricuță”, “Iuliu Hațieganu” University of Medicine and Pharmacy, 8, Victor Babeș Street, 400012 Cluj-Napoca, Romania; ancasbojan@yahoo.ca (A.S.B.); ciprian.tomuleasa@gmail.com (C.T.); deli_dima@yahoo.com (D.D.); m.zdrenghea@yahoo.com (M.Z.); 3 Department of Medical Informatics and Biostatistics, “Iuliu Hațieganu” University of Medicine and Pharmacy, 8, Victor Babeș Street, 400012 Cluj-Napoca, Romania; miancu@umfcluj.ro; 4 Department of Pharmacology and Toxicology, “Iuliu Hațieganu” University of Medicine and Pharmacy, 8, Victor Babeș Street, 400012 Cluj-Napoca, Romania; raluca_parlog@yahoo.com; 5 Department of Genetics, The Oncology Institute “Ion Chiricuță”, 34-36 Republicii Street, 400015 Cluj-Napoca, Romania; stefiipopa@yahoo.com (Ș.G.-P.); simona.visan19@gmail.com (S.V.); 6 Department of Medical Genetics, “George Emil Palade” University of Medicine, Pharmacy, Science and Technology, 38, Gheorghe Marinescu Street, 540139 Târgu-Mureș, Romania; tripon.florin.2010@gmail.com (F.T.); adriana.cosma@umfst.ro (A.S.C.); 7 Center for Advanced Medical and Pharmaceutical Research, “George Emil Palade” University of Medicine, Pharmacy, Science and Technology, 38, Gheorghe Marinescu Street, 540139 Târgu-Mureș, Romania; 8 Department of Pathology, The Oncology Institute “Ion Chiricuță”, 34-36 Republicii Street, 400015 Cluj-Napoca, Romania; feticab@yahoo.com; 9 Department of Medical Genetics, “Iuliu Hațieganu” University of Medicine and Pharmacy, 8, Victor Babeș Street, 400012 Cluj-Napoca, Romania; orsigaal92@gmail.com (I.O.G.); andreeamanuelamirea@gmail.com (A.-M.M.); radupopp2001@yahoo.com (R.A.P.); ivpop.cj.ro@gmail.com (I.V.P.); 10 Department of Community Medicine, “Iuliu Hațieganu” University of Medicine and Pharmacy, 8, Victor Babeș Street, 400012 Cluj-Napoca, Romania; miraflorea17@gmail.com; 11 Department of Therapeutic Chemistry, “Iuliu Hațieganu” University of Medicine and Pharmacy, 8, Victor Babeș Street, 400012 Cluj-Napoca, Romania; araniciu.catalin@umfcluj.ro; 12 Department of Cardiology, “Victor Babeș” University of Medicine and Pharmacy, 2, Eftimie Murgu Square, 300041 Timișoara, Romania; petrescu_lucian@yahoo.com

**Keywords:** myeloproliferative neoplasms, genetic predisposition, *TET2*, single nucleotide polymorphisms

## Abstract

Background: The complexity of myeloproliferative neoplasms (MPNs) cannot be characterized by acquired somatic mutations alone. Individual genetic background is thought to contribute to the development of MPNs. The aim of our study was to assess the association between the *TET2* rs1548483 single nucleotide polymorphism (SNP) and the susceptibility to polycythemia vera (PV), essential thrombocythemia (ET), primary myelofibrosis (PMF) or chronic myeloid leukemia (CML). Methods: We evaluated the *TET2* rs1548483 SNP through real-time PCR in 1601 MPN patients out of which 431 with PV, 688 with TE, 233 with PMF, 249 with CML and 197 controls. We included only patients with a molecularly proven driver mutation, such as *JAK2* V617F, *CALR* or *BCR-ABL1*. Results: Significant association between *TET2* rs154843 variant allele and *JAK2* V617F-positive PV and PMF (OR = 1.70; 95% CI: 1.01–2.91; *p*-value = 0.046, and OR = 2.04; 95% CI: 1.10–3.77; *p*-value = 0.024, respectively), and type 2 *CALR*-positive PMF (OR = 2.98; 95% CI: 1.12–7.93; *p*-value = 0.035) was noted. Conclusions: The *TET2* rs1548483 SNP is associated with the susceptibility to molecularly annotated PV and PMF.

## 1. Introduction

Myeloproliferative neoplasms (MPN) are a group of heterogeneous, clonal, stem-cell-derived disorders, characterized by the production of mostly mature appearing cells within the blood stream [1].

The concept of myeloproliferative disorders was first described by Dameshek in 1951. Since then, the term “myeloproliferative neoplasms” was introduced in 2008, suggesting the clonal nature of these disorders, followed by the 2016 revision of the WHO Classification of Tumours of Haematopoietic and Lymphoid Tissues which included the following as MPNs: chronic myeloid leukemia (CML); chronic neutrophilic leukemia; polycythemia vera (PV); primary myelofibrosis (PMF); essential thrombocythemia (ET); chronic eosinophilic leukemia, not otherwise specified; and MPN, unclassifiable [2,3,4].

The most common MPNs are PV, ET and PMF, grouped together, due to overlapping features, into the category of *BCR-ABL*-negative classical MPNs, and CML, the only *BCR-ABL*-positive MPN [5,6].

In the majority of *BCR-ABL*-negative neoplasms, specific somatic driver mutations have been described. These are mutually exclusive, interfering with the JAK-STAT pathway activation, and include *JAK2* (Janus kinase 2), *CALR* (calreticulin) and *MPL* (myeloproliferative leukemia virus oncogene). PV is almost invariably associated with mutations in the *JAK2* gene, the most common being *JAK2* V617F, which is also present in about 50–65% of patients with ET and PMF. *CALR* and *MPL* mutations are usually absent in PV. *CALR* mutations have a frequency of 20% to 25% in patients with ET and PMF, while *MPL* mutations have a frequency of 3–4% in ET and 6–7% in PMF [5,7]. The genetic landscape of MPNs is more complex than initially thought and involves mutations in several other genes beyond the three main driver ones. Recent sequencing studies revealed that mutations other than *JAK2*, *CALR*, or *MPL* are found in 81% of patients with PMF, 53% with PV, and 53% with ET. The most frequent additional genes that acquire molecular abnormalities in MPN patients are *TET2* (22% in PV, 18% in PMF and 16% in ET) and *ASXL1* (36% in PMF, 12% in PV and 11% in ET) [8,9].

In additions, *TET2* mutations have been described alongside *JAK2* mutations and others like *DNMT3A* or *ASXL1* mutations in age-related clonal hematopoiesis of indeterminate potential (CHIP), contributing to an increased risk of hematologic cancer development. According to a study by Jaiswal et al. assessing CHIP associated with adverse outcomes, the risk of hematologic cancers increased 11.1-fold in persons with a CHIP-associated mutation. This risk increased further to 49-fold among persons with a variant allele fraction of 0.10 or greater [10].

Ten-eleven translocation 2 *(TET2*) is part of the TET family and is a tumor suppressor gene that is inactivated in a wide range of hematological malignancies. The *TET2* enzyme, a methylcytosine dioxygenase, converts 5-methylcytosine (5-mC) into 5-hydroxymethylcytosine (5-hmC), leading to excision repair and replacement by an unmethylated cytosine, resulting in DNA demethylation and gene activation [11].

The *TET2* protein has a key role in the epigenetic regulation of gene expression during embryogenesis, differentiation of hematopoietic cells, cancer development, and it is involved in somatic cell reprogramming [12,13,14,15,16].

Somatic mutations in the gene *TET2* were originally described in 2009 by Delhommeau et al., and Langemeijer et al. They identified frequent somatic mutations in *TET2* in MPNs, myelodysplastic syndromes (MDS) and acute myeloid leukemia (AML) [17,18].

The vast spectrum of phenotypes seen in MPNs cannot be characterized by acquired somatic mutations alone. The individual genetic background is thought to contribute to the pathogenic mechanism involved in the development of MPNs.

In 2008, a Swedish large population-based case–control study, involving over 11.000 MPN patients and their first-degree relatives, showed a five- to sevenfold elevated risk of MPNs among the first-degree relatives of MPN patients. These findings support the hypothesis that inherited genetic variants can predispose to the acquisition of genetic mutations leading to uncontrollable proliferation of myeloid lineages [19].

Greater access to genome-scale molecular techniques also allowed a more detailed analysis of common single nucleotide polymorphisms (SNPs). This genetic landscaping allowed the identification of germline SNPs that provided more evidence of an inherited cause for MPN, such as *JAK2* 46/1 or GGCC haplotype, and various other SNPs in genes such as *TERT*, *MECOM*, *SH2B3*, *TET2*, *ATM*, *CHEK2*, *THRB-RARB*, *PINT* [20,21,22,23,24,25,26,27].

Not only are *TET2* somatic mutations frequent events in MPN and CHIP, but also the constitutional genetic variation at *TET2* locus seems to play a role in MPN predisposition. Hinds et al. performed a genome-wide association study (GWAS) to identify additional germline risk factors associated with MPNs. In this study they described a SNP in *TET2* (rs1548483 C > T) nominally associated with *BCR-ABL* negative MPNs, as well as CML and systemic mastocytosis (SM) [27].

These examples offer a unique insight into the underlying etiology of myeloid malignancies and present opportunities to understand disease occurrence and the reasons behind the vast phenotypically heterogeneity in these patients. To address this issue, we have analyzed a large cohort of MPN patients, aiming to establish the additional contribution of the recently described *TET2* rs1548483 SNP to the occurrence of MPN phenotypes and their associated somatic mutations.

## 2. Materials and Methods

### 2.1. Research Ethics Considerations 

This study was conducted in accordance with the principles of the 1975 Declaration of Helsinki and approved by the Ethics Committee of Iuliu Hațieganu University of Medicine and Pharmacy, Cluj-Napoca, Romania. Each participant of the study has given a written consent regarding genetic testing.

### 2.2. Patients and Controls

A total of 1601 patients diagnosed with MPN were included in this study: 431 with PV, 688 with TE, 233 with PMF and 249 with CML. The patients were diagnosed between 1984 and 2019 in various hematology departments from hospitals in Romania. All patients were diagnosed or reclassified according to the latest WHO classification of myeloid neoplasms [6]. Demographical data as well as distribution of driver mutations in MPN patients included in the study are presented in Table 1. The study included only patients with a molecularly proven driver mutation, *JAK2* V617F, *CALR* or *BCR-ABL1.* Patients with ET or PMF with *MPL* mutations were ruled out from inclusion in this study, due to their low number. ET and PMF patients who did not harbor any *JAK2* V617F, *CALR* or *MPL* mutations, also known as “triple-negative”, were not considered of being included in this study. Because of the lack of bone marrow biopsies from these patients and MPN-associated additional mutation status, their diagnosis was considered uncertain. Among the 1601 included patients, 939 patients were explored in our previous work for other germ-line variants. Specifically 454 patients with ET, 337 patients with PV and 148 patients with PMF had been previously genotyped for *TERT* rs2736100, *MECOM* rs2201862, *HBS1L-MYB* rs9376092, and *THRB-RARB* rs4858647 polymorphisms and *JAK2* 46/1 haplotype [24]. A total of 884 patients, namely 427 with ET, 316 with PV, and 139 PMF were successfully genotyped for all SNPs—the five above-mentioned ones and *TET2* rs1548483. 

The study also included 197 individuals representing the control group. Only individuals with no hematological malignancy were included in the control group. Individuals from the control group were referred for routine blood workup to the Hematology Clinic from Cluj-Napoca, Romania. 

The age distribution differed significantly between MPN subtypes, the median age values of MPN patients with different subtypes and control subjects being 64 years (range: 29–89 years) for the PV group, 60 years (range: 19–91 years) for the ET group, 66 years (range: 27–93 years) for the PMF group, 54 years (range: 18–85 years) for the CML and 30 years (range: 26–63 years) for the control subjects, respectively (*p* < 0.001). Gender distribution was unbalanced between MPN subtypes and controls (*p* < 0.001), thus female gender being more frequent between ET patients (male: female ratio = 1.7) while males were more frequent in the PV group (Table 1). 

### 2.3. Genotyping Methods

Genomic DNA was isolated from whole blood samples collected on EDTA, from all participants, using various commercial kits (Wizard Genomic DNA Purifcation kit, Promega, Madison, WI, USA; Quick gDNA MiniPrep kit, Zymo Research, Irvine, CA, USA; PureLink Genomic DNA Mini Kit, Invitrogen, Thermo Fisher, Waltham, MA, USA). Somatic mutations analyzed in patients, namely *JAK2* V617F, *CALR* exon 9 indels and *BCR-ABL1*, were detected at diagnosis or at the moment when the particular technique became available in our center. *JAK2* V617F mutation was assessed using a tetra-primer PCR assay until 2015, and thereafter using a real-time PCR assay [28,29]. *CALR* exon 9 indels were analyzed using a simplex PCR [30]. For CML patients, *BCR-ABL1* major transcript was assessed at diagnosis using a qualitative nested PCR [31]. Quantification of *BCR-ABL1* was evaluated whenever necessary with an automated, cartridge-based real-time PCR system (BCR-ABL Ultra, GeneXpert system, Cepheid, Sunnyvale, CA, USA). 

*TET2* rs1548483 SNP was genotyped in all patients and controls using a TaqMan 5 exonuclease assay (assay number C___7512138_20), as recommended by the manufacturer (Applied Biosystems, Thermo Fisher, Waltham, MA, USA), using Quant Studio 3 or 7500 Fast Dx real-time PCR systems (Applied Biosystems, Thermo Fisher, Waltham, MA, USA). 

### 2.4. Statistical Analysis

Data for nominal variables were presented as number cases and percentage (relative frequencies), while the median with interquartile range [lower quartile; upper quartile] was used for describing distributions of continuous variables. The nonparametric Mann–Whitney U test was used to compare the age distribution between MPN cases and controls. 

The null hypothesis that the Hardy–Weinberg equilibrium holds in MPN cases was evaluated using an exact chi-square test. The chi-square test was also used to evaluate the genotype frequency distribution between MPN subtypes and control group.

The independent contribution of *TET2* SNP to odds of MPN phenotypes was assessed by multiple multinomial logistic regression analysis. Regression analysis was applied under codominant, dominant, overdominant and recessive genetic models using the SNPassoc R package [32]. Association between *TET2* SNP and each MPN subtype was measured by the odds ratio (OR) and 95% confidence interval (CI) with adjustment for age and gender. 

We also looked for epistatic interactions between the studied *TET2* SNP and other SNPs (the *JAK2* 46/1 haplotype-tagging *JAK2* rs10974944, *TERT* rs2736100, *MECOM* rs2201862, *HBS1L-MYB* rs9376092, and *THRB-RARB* rs4858647). These epistatic interactions were analyzed by SNPassoc package for R using a loglikelihood ratio test (LRT) assuming codominant, dominant, recessive and overdominant models.

A post hoc power analysis was also performed using genpwr R package using minor allele frequency of studied TET2 rs1548483 SNP, odds ratio and empirical sample size as input parameters [33]. Post hoc analysis showed that we had 80.3% power (1-β) to detect an odds ratio of 2 in a sample size of 1798 patients including 197 controls and 1601 cases (case rate of 89%) over an average minor allele frequency of 0.07 for TET2 rs1548483 SNP in MPN population and assuming an alpha of 0.05 and an additive genetic model. The estimated power values for each MPN subtype were between 70% (for CML) and 94% (for PMF) for the same specified parameters. 

All statistical analysis was performed using R software [34]. The statistical tests were two-sided, and a result with *p* < 0.05 was considered statistically significant. In the case of hypothesis testing on the four genetic models, the false discovery rate *p*-value adjustment based on Benjamini and Hochberg procedure was reported as corrected *p*-value in the case of logistic models with significant results. 

## 3. Results

### 3.1. Association between TET2 rs154843 SNP and MPN Subtypes—Allelic Model

The basic information concerning genotype distribution, minor allele frequency (MAF) in studied MPN subtypes and control group are summarized in Table 2. The genotype distribution of *TET2* SNP satisfied the Hardy–Weinberg equilibrium in the analyzed reference population (*p*-value = 0.334 for the control group).

Out of the analyzed patient groups, the PV and PMF groups showed a significant increase of MAF compared to control group (OR = 1.70; 95% CI: 1.01–2.91; *p*-value = 0.046, and OR = 2.02; 95% CI: 1.14–3.57; *p*-value = 0.015, respectively). Taking into consideration molecular subtypes, *TET2* variant allele was associated with *JAK2* V617F-positive PV and PMF (OR = 1.70; 95% CI: 1.01–2.91; *p*-value = 0.046, and OR = 2.04; 95% CI: 1.10–3.77; *p*-value = 0.024, respectively). The association between *TET2* variant allele and *CALR*-positive PMF bordered the statistical significance (OR = 1.95; 95% CI: 0.95–4.02; *p*-value = 0.066), on the behalf of *CALR* type 2 mutations (OR = 2.98; 95% CI: 1.12–7.93; *p*-value = 0.035). Table 2 shows the detailed results regarding the analysis of *TET2* MAF in various MPN subtypes, compared to control group.

### 3.2. Association between TET2 rs154843 SNP and MPN Phenotypes—Genotypic Models

The variant genotypes of *TET2* SNP were significantly associated with an increased risk of PMF in the codominant and overdominant models tested (OR = 2.4; 95% CI: 1.3–4.43; *p*-value = 0.005; corrected *p*-value = 0.013 and OR = 2.41; 95% CI: 1.31–4.45; *p*-value = 0.005, corrected *p*-value = 0.0125 respectively). *TET2* variant genotypes remained an independent risk factor also after adjusting for age and sex (OR = 1.95, 95% CI: 1.02–3.73; *p*-value = 0.044 and OR = 1.96; 95% CI: 1.03–3.76; *p*-value = 0.041, respectively). In addition, there was a significant positive association between variant genotype of *TET2* SNP and risk of PMF in the dominant model (OR = 2.26; 95% CI: 1.24–4.12; *p*-value = 0.008; corrected *p*-value = 0.0133), this association bordering the statistical significance after the adjustment for age and sex (OR = 1.84; 95% CI: 0.97–3.47; *p*-value = 0.062). Also, a tendency toward statistical significance was noticed for the association with PV risk under the dominant model (OR = 1.68; 95% CI: 0.95–2.96; *p*-value = 0.074). However, this association was no longer observed after the adjustment for age and sex (*p*-value > 0.05). Table 3 shows in detail the results regarding the association between *TET2* SNP and MPN phenotypes, using different genotypic models.

### 3.3. Association between TET2 rs154843 SNP and MPN Molecular Subtypes—Genotypic Models

#### 3.3.1. *JAK2* V617F Mutation

We observed positive association between the variant genotypes of *TET2* SNP and *JAK2* V617F-positive PMF in the codominant, dominant and overdominant models tested (OR = 2.43; 95% CI: 1.26–4.69; *p*-value = 0.008; corrected *p*-value = 0.020; OR = 2.29; 95% CI: 1.2–4.37; *p*-value = 0.012; corrected *p*-value = 0.020 and OR = 2.44; 95% CI: 1.26–4.72; *p*-value = 0.008; corrected *p*-value = 0.020 respectively). This association became weaker after adjustment for age and gender, bordering the statistical significance in this case for all three models (OR = 1.99; 95% CI: 0.98–4.07; *p*-value = 0.056; OR = 1.88; 95% CI: 0.94–3.79; *p*-value = 0.076 and OR = 2.01; 95% CI: 0.99–4.01; *p*-value = 0.053, respectively). Also in the case of *JAK2* V617F-positive PV, a near significant association was observed in the dominant model (crude OR = 1.68; 95% CI: 0.95–2.96; *p*-value = 0.074). However, this association was no longer observed after the adjustment for age and sex (*p*-value > 0.05).

Table 4 shows in detail the results regarding the association between *TET2* SNP and different *JAK2* V617F-positive MPN, using different genotypic models.

#### 3.3.2. *CALR* Mutations

The results highlighted a positive significant association between the variant genotype of *TET2* SNP and risk of *CALR*-positive PMF in the dominant and overdominant inheritance models tested (OR = 2.18; 95% CI: 1.02–4.66; *p*-value = 0.045 corrected *p*-value = 0.075 and OR = 2.33; 95% CI: 1.08–5.03; *p*-value = 0.031; corrected *p*-value = 0.075). However, this association was no longer observed after the adjustment for age and sex (*p*-value > 0.05). We then explored the possible different effect of *TET2* SNP on the two major types of *CALR* mutations, type 1 and type 2, respectively. No statistical associations were observed between *TET2* SNP and *CALR* type 1 mutations in any MPN subtype (*p*-value > 0.05 for all these comparisons). However, in the case of type 2 mutations, the association was significant in PMF in codominant, dominant and overdominant models (OR = 3.75; 95% CI: 1.30–10.78; *p*-value = 0.014; corrected *p*-value = 0.030; OR = 3.53; 95% CI: 1.24–10.08; *p*-value = 0.018; corrected *p*-value = 0.030, and OR = 3.77; 95% CI: 1.31–10.84; *p*-value = 0.014; corrected *p*-value = 0.030, respectively). After the adjustment for age and sex, the associations were no longer significant (*p*-value > 0.05). 

Table 5 describes in detail the results regarding the association between *TET2* rs154843 and different *CALR*-positive MPNs, using different genotypic models.

### 3.4. Epistatic Two-Way SNPs Interaction Stratified by MPN Subtypes

We also tested for epistatic interaction between *TET2* rs154843 and other SNPs (*TERT* rs2736100, *MECOM* rs2201862, *HBS1L-MYB* rs9376092, and *THRB-RARB* rs4858647), which we previously genotyped in PV, ET and PMF (Table 5). Based on the log-likelihood ratio test (LRT), we identified epistatic interactions between *TET2* rs154843 and *HBS1L-MYB rs9376092* in PV (p_interaction_ = 0.012 under overdominant model). We also identified epistatic interactions between *TET2* rs154843 and *HBS1L-MYB rs9376092* (p_interaction_ = 0.014 and p_interaction_ = 0.049 under codominant and overdominant model) and *JAK2* rs10974944, which tags the *JAK2* 46/1 haplotype (p_interaction_ = 0.037 under recessive model) in ET. We found no significant epistatic SNPs interaction between *TET2* rs154843 and other SNP on susceptibility to PMF in none of the inheritance genetic models (Table 6). 

## 4. Discussion

To validate our supposition that a germ-line variant like *TET2* rs1548483 polymorphism is associated with the development of MPN, we conducted this observational study and demonstrated that there was a significant association between the variant allele of the *TET2* polymorphism and PV and PMF risk in our MPN patients. This observation parallels data reported by Hinds et al., who performed a GWAS and identified *TET2* rs1548483 SNP among others, to associate with various *BCR-ABL1* negative MPNs and *JAK2* V617 clonal hematopoiesis in general population. In their study, *TET2* rs1548483 SNP had the fourth strongest contribution to the occurrence of MPN, after *JAK2* rs5938477, *TERT* rs7705526, *SH2B3* rs7310615. Although *TET2* rs1548483 SNP was not significantly associated with V617F status in MPN cases, there was a trend towards larger effect sizes for *JAK2* V617F-positive than for V617F-negative patients [27]. However, they did not report on *CALR*-positive MPN, which represents a major molecular subtype of ET and PMF. On the contrary, our study outlined not only the association between *TET2* rs1548483 and each MPN phenotypes, but also the two major molecular subtypes seen in PV, ET and PMF, namely *JAK2* V617F and *CALR*. In this respect, we noted significant association between *TET2* rs1548483 variant allele and *JAK2* V617F-positive PV and PMF. Additionally, in the PMF group, this association bordered the statistical significance in the case of *CALR* mutations. Interestingly, this association was largely on behalf of type 2 *CALR* mutations. This might suggest different consequences of the *TET2* rs1548483 SNP on acquiring various *CALR* mutations. Due to the fact that this study did not include patients with *MPL* mutations or ”triple-negative” patients, we acknowledge a possible selection bias, especially in case of the PMF subgroup, in which *MPL* mutations account for about 6–7% and ”triple-negative” PMF patients for about 15% [5]. In further studies we plan on investigating the stability of the effect size (OR) through a sensitivity analysis on all PMF patients.

Other germ-line SNPs at the *TET2* locus have been described in recent publications. Shen et al. found that *TET2* rs3733609 C/T variant genotypes had higher incidence in *JAK2* V617F-positive sporadic MPN patients. Similarly, Xiao et al. described the same SNP in *JAK2* V617F-positive MPN patients, reporting a higher frequency of the *TET2* SNP in *JAK2* V617F high allele burden group than in the low allele burden group, suggesting that its presence may influence clinical characteristics and clonal evolution of MPN patients [35,36].

A study on Egyptian CML patients noted that rs3442524, one out of three studied *TET2* SNPs (rs2454206, rs34402524, rs61744960) was associated with larger spleen size and higher *BCR-ABL1* levels after six months of starting treatment with tyrosine kinase inhibitors, without altering the prognostic criteria of patients. Looking into CML patients, the results of Hinds et al. GWAS study nominally associated *TET2* rs154843 with CML. However, we did not see any association between *TET2* rs1548483 SNP and CML [27,37]. 

Besides different SNPs, Hinds et al. also replicated the *JAK2* 46/1 haplotype, a germline haplotype, commonly referred to as “46/1” or “GGCC” situated on chromosome 9p, present in about 45% of the general population [38]. Reports described that subjects who were heterozygotes for this haplotype were more likely to acquire the *JAK2* V617F mutation with the predisposition SNP allele than on the other chromosome, attributing to subjects a three to four times higher risk of developing an *JAK2*-positive MPN. Correlations with other MPN driver mutations besides *JAK2* V617F have not yet been firmly confirmed [39].

As far as germ-line variants are concerned, our study observed a statistical significant interaction between the recessive genetic inheritance model of *TET2* polymorphism and the *JAK2* 46/1 haplotype in ET patients. In ET patients as well as PV patients we identified epistatic interactions between *TET2* rs154843 and *HBS1L-MYB* rs9376092. Interactions between specific germ-line variants may possibly influence the risk of developing MPN, and may contribute to the clinical course and clonal evolution of MPN patients. However, it should be noted that the results regarding the SNP–SNP epistatic interactions were obtained using a statistics-only approach. This statistical approach does not necessarily reflect the biological insights of such an epistatic interaction. For instance, the *HBS1L-MYB* rs9376092 SNP was shown to reduce *MYB* expression in normal myeloid cells [26]. Caution should be exercised when looking at the results we obtained regarding the epistatic interaction between *TET2* and *HBS1L-MYB* SNPs, as we were not able to perform methylation and expression studies. Further studies investigating interactions between germ-line mutations are needed to confirm this supposition. 

The present study expands our previous work regarding the genetic predisposition to MPN. Our previous work reported significant association between *TERT* rs2736100 and *MECOM* rs2201862, and MPN, regardless of molecular subtype, *HBS1L-MYB* rs9376092 and *JAK2* V617F-positive ET, *THRB*-*RARB* rs4858647 and PMF, *SH2B3* rs3184504 and *JAK2* V617F-positive MPN. We also replicated the *JAK2* 46/1 haplotype in previous work, confirming the association with *JAK2* V617F-positive MPNs [23,24,40]. By showing significant associations between *TET2* rs154843 SNP and various MPN phenotypes, such as PV and PMF, and molecular subtypes, such as *JAK2* V617F-positive PV and PMF, type 2 *CALR*-positive PMF, we believe our study contributes to the addition of important data regarding the genetic landscape of MPN patients. 

The most important strength of our study is the large number of patients included, contributing to the statistical relevance of our results. Also, the fact that all patients had information regarding main somatic driver mutations, greatly adds to the strength of this study, enabling us to demonstrate differential effects of *TET2* rs154843 SNP on various molecular MPN subtypes. 

Although a significant proportion of the patients had information regarding several other germline variants, we consider that a limitation of our study was the lack of data regarding other polymorphisms at the genomic level that could interact with *TET2* rs154843 SNP. In addition, it would have been useful to characterize the profile of *TET2* somatic mutations in patients included in our study, in order to search for possible correlations between *TET2* rs154843 SNP and the acquisition of *TET2* somatic mutations. Unfortunately, we were not able to perform *TET2* somatic mutation analysis. Moreover, we were not able to compare the methylation pattern at the *TET2* locus between the three genotypes groups (CC, CT and TT) and to analyze the functional consequences of the *TET2* SNP. These data could help in verifying especially the overdominant effects of *TET2* SNP observed in this study. Also, due to insufficient clinical data, we were not able to analyze the influence of the *TET2* rs154843 SNP on clinical outcomes of patients and phenotype.

## 5. Conclusions

In conclusion, we report a significant association between *TET2* rs154843 and PV and PMF phenotypes. Moreover, *TET2* rs154843 associates with *JAK2* V617F-positive PV and PMF, and type 2 *CALR*-positive PMF. Future studies should focus on the complex interplay between multiple polymorphisms and their functional consequences related to MPN susceptibility and disease outcome.

## Figures and Tables

**Table 1:** Distribution of demographic characteristics and phenotypic driver mutations in MPN patient subgroups.

Variable	MPN Subtypes			
	PV (*n*_1_ = 431)	ET (*n*_2_ = 688)	PMF (*n*_3_ = 233)	CML (*n*_4_ = 249)
Male gender, *n* (%)	222 (51.5)	255 (37.1)	112 (48.1)	125 (50.2)
Age at diagnosis, years; median [Q_1_; Q_3_]	64 [57; 71]	60 [48; 70]	66 [57; 73]	54 [43; 64]
*JAK2* V617F+, *n* (%)	431 (100)	525 (76.3)	151 (64.8)	0 (0.0)
*CALR*+, *n* (%)	0 (0.0)	163 (23.7)	82 (35.2)	0 (0.0)
*CALR* type 1+, *n* (%)	0 (0.0)	107 (15.4)	56 (24.0)	0 (0.0)
*CALR* type 2+, *n* (%)	0 (0.0)	56 (8.1)	26 (10.3)	0 (0.0)
*BCR-ABL1* fusion; *n* (%)	0 (0.0)	0 (0.0)	0 (0.0)	249 (100)

*n* = absolute frequency; Q_1_ = lower quartile; Q_3_ = upper quartile.

**Table 2:** *TET2* rs154843 SNP (single nucleotide polymorphism) distribution in control group and MPN (myeloproliferative neoplasm) subtypes.

	*TET2* Genotypes			MAF ^a^ [95% CI]	p_HWE_^b^	p_allelic_^c^	OR [95% CI]
	**CC**	**CT**	**TT**				
Controls (*n* = 197)	180 (91.4)	16 (8.1)	1 (0.5)	4.57 [2.73; 7.12]	0.3340	-	Reference
PV (*n* = 431)	372 (86.3)	53 (12.3)	6 (1.4)	7.54 [5.87; 9.51]	0.0251	0.046 *	1.70 [1.01; 2.91] *
ET (*n* = 688)	609 (88.5)	76 (11.0)	3 (0.4)	5.96 [4.77; 7.34]	0.7272	0.294	1.32 [0.78; 2.23]
PMF (*n* = 233)	192 (82.4)	41 (17.6)	0 (0.0)	8.80 [6.39; 11.75]	0.2297	0.015 *	2.02 [1.14; 3.57] *
CML (*n* = 249)	225 (90.4)	24 (9.6)	0 (0.0)	4.82 [3.11; 7.09]	1.0000	0.867	1.06 [0.57; 1.98]
*JAK2* V617F^+^ PV (*n* = 431)	372 (86.3)	53 (12.3)	6 (1.4)	7.54 [5.87; 9.51]	0.0251	0.046 *	1.70 [1.01; 2.91] *
*JAK2* V617F^+^ ET (*n* = 525)	459 (87.4)	63 (12.0)	3 (0.6)	6.57 [5.15; 8.24]	0.4821	0.152	1.47 [0.87; 2.50]
*JAK2* V617F^+^ PMF (*n* = 152)	125 (82.2)	27 (17.8)	0 (0.0)	8.88 [5.93; 12.66]	0.6076	0.024 *	2.04 [1.10; 3.77] *
*CALR*^+^ ET (*n* = 163)	150 (92.0)	13 (8.0)	0 (0.0)	3.99 [2.14; 6.72]	1.0000	0.711	0.87 [0.42; 1.80]
*CALR* type 1^+^ ET (*n* = 106)	100 (94.3)	6 (5.7)	0 (0.0)	2.83 [1.05; 6.06]	1.0000	0.295	0.61 [0.24; 1.56]
*CALR* type 2^+^ ET (*n* = 56)	49 (87.5)	7 (12.5)	0 (0.0)	6.25 [2.25; 12.45]	1.0000	0.622	1.39 [0.57; 3.42]
*CALR*^+^ PMF (*n* = 82)	68 (82.9)	14 (7.1)	0 (0.0)	8.54 [4.75; 13.91]	1.0000	0.066	1.95 [0.95; 4.02]
*CALR* type 1^+^ PMF (*n* = 56)	48 (85.7)	8 (14.3)	0 (0.0)	7.14 [3.13; 13.59]	1.0000	0.276	1.61 [0.68; 3.80]
*CALR* type 2^+^ PMF (*n* = 24)	18 (75.0)	6 (25.0)	0 (0.0)	12.50 [4.73; 25.24]	1.0000	0.035 *	2.98 [1.12; 7.93] *
*BCR-ABL*^+^ CML (*n* = 249)	225 (90.4)	24 (9.6)	0 (0.0)	4.82 [3.11; 7.09]	1.0000	0.861	1.06 [0.57; 1.98]

Data were expressed as absolute frequencies and relative frequencies; ^a^ minor allele frequency represented as percentage; ^b^ *p*-values calculated from exact tests of Hardy–Weinberg equilibrium; ^c^ *p*-values were calculated from two-sided χ^2^ test; bold values denote significant results at the *p* < 0.05 level (marked with asterisk *).

**Table 3:** Association between *TET2* rs154843 SNP and MPN phenotypes: multinomial logistic regression results.

	PV				ET				PMF				CML			
	Crude OR [95% CI]	*p* ^+^	Adjusted OR ^a^ [95% CI]	*p* ^+^	Crude OR [95% CI]	*p* ^+^	Adjusted OR [95% CI]	*p* ^+^	Crude OR [95% CI]	*p* ^+^	Adjusted OR [95% CI]	*p* ^+^	Crude OR [95% CI]	*p* ^+^	Adjusted OR [95% CI]	*p* ^+^
Codominant Model																
CC	1		1		1		1		1		1		1		1	
CT	1.60 [0.89; 2.88]	0.113	1.29 [0.69; 2.42]	0.421	1.40 [0.80; 2.47]	0.238	1.19 [0.66; 2.14]	0.570	2.40 [1.30; 4.43] *	0.005 *	1.95 [1.02; 3.73] *	0.044 *	1.20 [0.62; 2.33]	0.589	1.07 [0.05; 2.09]	0.849
TT	2.90 [0.35; 4.28]	0.326	2.33 [0.22; 24.55]	0.477	0.89 [0.09; 8.57]	0.917	0.72 [0.06; 8.08]	0.794	0.0001 [NA]	0.913	0.0001 [NA]	0.964	0.0001 [NA]	0.911	0.0001 [NA]	0.952
Dominant Model																
CC	1		1		1		1		1		1		1		1	
CT + TT	1.68 [0.95; 2.96]	0.074	1.36 [0.74; 2.49]	0.327	1.37 [0.79; 2.38]	0.258	1.16 [0.65; 2.06]	0.615	2.26 [1.24; 4.12] *	0.008 *	1.84 [0.97; 3.47]	0.062	1.13 [0.59; 2.17]	0.714	1.01 [0.52; 1.95]	0.989
Recessive Model																
CC + CT	1		1		1		1		1		1		1		1	
TT	1.84 [0.20; 16.63]	0.587	2.64 [0.26; 26.55]	0.411	0.86 [0.09; 8.35]	0.898	1.05 [0.10; 10.88]	0.968	0.001 [NA]	0.819	0.001 [NA]	0.926	0.001 [NA]	0.817	0.001 [NA]	0.902
Overdominant Model																
CC + TT	1		1		1		1		1		1		1		1	
CT	1.59 [0.88; 2.85]	0.124	1.28 [0.68; 2.40]	0.438	1.40 [0.80; 2.47]	0.238	1.19 [0.66; 2.15]	0.565	2.41 [1.31; 4.45] *	0.005 *	1.96 [1.03; 3.76] *	0.041 *	1.21 [0.62; 2.34]	0.579	1.07 [0.55; 2.11]	0.834

^+^*p*-values obtained from Wald test of multinomial logistic regression comparing each group with the control group (reference category); ^a^ adjusted for age group (>60 years versus ≤60 years) and sex (M versus F) in multinomial logistic model; NA = not available because of null frequencies; bold values denote significant results at the *p* < 0.05 level (marked with asterisk *).

**Table 4:** Associations between *TET2* rs154843 SNP and MPN molecular subtypes determined by *JAK2* V617F mutation: multinomial logistic regression results.

	*JAK2* V617F ^+^ PV				*JAK2* V617F ^+^ ET				*JAK2* V617F ^+^ PMF			
	Crude OR [95% CI]	*p* ^+^	Adjusted OR ^a^ [95% CI]	*p* ^+^	Crude OR [95% CI]	*p* ^+^	Adjusted OR [95% CI]	*p* ^+^	Crude OR [95% CI]	*p* ^+^	Adjusted OR [95% CI]	*p* ^+^
Codominant Model												
CC	1		1		1		1		1		1	
CT	1.60 [0.89; 2.88]	0.115	1.32 [0.70; 2.52]	0.390	1.55 [0.87; 2.75]	0.138	1.33 [0.72; 2.4]	0.360	2.43 [1.26; 4.69] *	0.008 *	1.99 [0.98; 4.07]	0.056
TT	2.94 [0.35; 24.87]	0.322	2.38 [0.23; 2.46]	0.468	1.19 [0.12; 11.64]	0.880	0.96 [0.09; 10.74]	0.975	0.002 [NA]	0.819	0.001 [NA]	0.897
Dominant Model												
CC	1		1		1		1		1		1	
CT + TT	1.68 [0.95; 3.03]	0.074	1.39 [0.75; 2.59]	0.301	1.52 [0.87; 2.67]	0.141	1.31 [0.72; 2.38]	0.376	2.29 [1.20; 4.37] *	0.012 *	1.88 [0.94; 3.79]	0.076
Recessive Model												
CC + CT	1		1		1		1		1		1	
TT	1.84 [0.20; 16.55]	0.588	2.79 [0.26; 9.04]	0.392	0.37 [0.02; 6.02]	0.488	0.51 [0.03; 9.04]	0.649	0.001 [NA]	0.923	0.004 [NA]	0.808
Overdominant Model												
CC + TT	1		1		1		1		1		1	
CT	1.59 [0.88; 2.85]	0.123	1.31 [0.69; 2.49]	0.406	1.54 [0.87; 2.74]	0.140	1.33 [0.72; 2.46]	0.359	2.44 [1.26; 4.72] *	0.008 *	2.01 [0.99; 4.10]	0.053

^+^*p*-values obtained from Wald test of multinomial logistic regression comparing each group with the control group (reference category); ^a^ adjusted for age group (>60 years versus ≤60 years) and sex (M versus F) in multinomial logistic model; NA = not available because of null frequencies; bold values denote significant results at the *p* < 0.05 level (marked with asterisk *).

**Table 5:** Associations between *TET2* rs154843 SNP and *CALR*-positive MPN molecular subtypes: multinomial logistic regression results.

	*CALR*^+^ ET		*CALR* Type 1^+^ ET		*CALR* Type 2^+^ ET		*CALR*^+^ PMF		*CALR* Type 1^+^ PMF		*CALR* Type 2^+^ PMF	
	Crude OR [95% CI]	Adjusted OR ^a^ [95% CI]	Crude OR [95% CI]	Adjusted OR [95% CI]	Crude OR [95% CI]	Adjusted OR [95% CI]	Crude OR [95% CI]	Adjusted OR [95% CI]	Crude OR [95% CI]	Adjusted OR [95% CI]	Crude OR [95% CI]	Adjusted OR [95% CI]
Codominant Model												
CC	1	1	1	1	1	1	1	1	1	1	1	1
CT	0.98 [0.45; 2.09]	0.58 [0.46; 3.29]	0.68 [0.26; 1.78]	0.44 [0.14;1.43]	1.61 [0.63;4.12]	0.95 [0.27; 3.37]	2.32 [1.07; 5.00]	1.23 [0.46; 3.29]	1.88 [0.76; 4.64]	1.17 [0.37;3.65]	**3.75** [1.30; 10.78] *	1.71 [0.36; 8.07]
TT	0.003 [NA]	0.004 [NA}	0.0001 [NA]	0.0001 [NA]	0.002 [NA]	0.0001 [NA]	0.01 [NA]	0.01 [NA]	0.0001 [NA]	0.0001 [NA]	0.03 [NA]	0.0001 [NA]
Dominant Model												
CC	1	1	1	1	1	1	1	1	1	1	1	1
CT + TT	0.92 [0.43; 1.95]	0.54 [0.21; 1.37]	0.64 [0.24; 1.66]	0.41 [0.13; 1.33]	1.51 [0.59;3.85]	0.89 [0.25; 3.14]	2.18 [1.02; 4.66] *	1.16 [0.44; 3.07]	1.76 [0.72; 4.33]	1.10 [0.36; 3.42]	3.53 [1.24; 10.08] *	1.61 [0.34; 7.51]
Recessive Model												
CC + CT	1	1	1	1	1	1	1	1	1	1	1	1
TT	2.44 [0.22; 27.20]	2.15 [0.14; 34.27]	1.97 [0.12; 30.22]	3.18 [0.20; 51.69]	3.57 [0.22; 58.05]	0.52 [0.01;171.66]	0.005 [NA]	0.0001 [NA]	0.005 [NA]	0.003 [NA]	0.008 [NA]	0.0001 [NA]
Overdominant Model												
CC + TT	1	1	1	1	1	1	1	1	1	1	1	1
CT	0.98 [0.46; 2.10]	0.58 [0.23; 1.47]	0.68 [0.26; 1.79]	0.44 [0.14; 1.43]	1.62 [0.63; 4.15]	0.96 [0.27; 3.39]	2.33 [1.08; 5.03] *	1.24 [0.46; 3.30]	1.88 [0.76; 4.67]	1.17 [0.38; 3.67]	3.77 [1.31;10.84] *	1.72 [0.37; 8.11]

Odds ratios estimated from multinomial logistic regression analysis comparing each group with the control group (reference category); ^a^ adjusted for age group (>60 years versus ≤60 years) and sex (M versus F) in multinomial logistic model; NA = not available because of null frequencies; bold values denote significant results at the *p* < 0.05 level; (marked with asterisk *).

**Table 6:** The *p*-values for two-dimensional interaction between *TET2* rs154843 SNP and other SNPs in MPN subtypes.

MPN Subtypes	Genetic Inheritance Models	*JAK2* rs10974944	*TERT* rs2736100	*HBS1L-MYB* rs9376092	*MECOM* rs2201862	*THRB-RARB* rs4858647
ET ^a^	Codominant	0.249	0.422	0.049 *	0.648	0.270
	Dominant	0.707	0.943	0.051	0.163	0.630
	Recessive	0.037 *	0.442	-	0.308	0.198
	Overdominant	0.667	0.743	0.014 *	0.504	-
	Codominant	0.725	0.969	0.093	0.951	0.301
PV ^b^	Dominant	0.259	0.370	0.696	0.437	0.166
	Recessive	0.496	0.830	-	0.671	-
	Overdominant	0.918	0.772	0.012 *	0.621	0.118
PMF ^c^	Codominant	0.711	0.341	0.374	0.687	0.857
	Dominant	0.676	0.681	0.244	0.712	0.862
	Recessive	-	-	-	-	-
	Overdominant	0.763	0.124	0.294	0.646	0.817

*p*-values for epistatic pairwise interactions obtained from the log-likelihood ratio test (LRT). ^a^*n* = 427 ET cases included in analysis; ^b^ *n* = 316 PV cases included in analysis; ^c^ *n* = 139 PMF cases included in analysis; bold values denote significant results at the *p* < 0.05 level (marked with asterisk *); all *p*-values are not adjusted for multiple comparisons.
